# Sonia delaunay: a life in the *avant*-*garde*

**DOI:** 10.1080/17571472.2016.1204009

**Published:** 2016-07-22

**Authors:** Francesco Carelli

**Affiliations:** ^a^ Professor of Family Medicine, EURACT Executive, Milano, Italy

Sonia Delaunay (1885–1979) was a key figure in the Parisian *avant*-*garde*, whose vivid and colourful work spanned painting, fashion and design. In 2015, Tate Modern in London presented the first UK retrospective to assess the breadth of her vibrant artistic career, from her early figurative painting in the 1900s to the energetic abstract work of the 1960s. The exhibition offered a radical reassessment of Delaunay’s importance as an artist, showcasing her originality and creativity across the twentieth century.

Born in Odessa and trained in Germany, Sonia Delaunay came to Paris in 1906 to join the emerging *avant*-*garde.* She met and married the artist Robert Delaunay, with whom she developed ‘*Simultaneism’* – a style of abstract compositions with dynamic contrasting colours and shapes.

Many iconic examples of these works were brought together at Tate Modern, including *Bal Bullier* [1913] and *Electric Prisms* [1914]. Her work expressed the energy of modern urban life, celebrating, for example, the birth of electric street lighting and the excitement of contemporary ballets and ballrooms.

The exhibition showed how the artist dedicated her life to experimenting with colour and abstraction, bringing her ideas off the canvas and into the world through tapestry, textiles, mosaic and fashion. Delaunay premiered her first ‘simultaneous dress’ of bright patchwork colours in 1913 and even opened a boutique in Madrid in 1918! Her *Atelier Simultané* in Paris went on to produce radical and progressive designs for scarves, umbrellas, hats, shoes and swimming costumes throughout the 1920s and 1930s. Clients included the Hollywood star Gloria Swanson and the architect Erno Goldfinger, as well as department stores like Metz & Co and Liberty.

The exhibition revealed how Delaunay’s designs presented her as a progressive woman synonymous with

Modernity: embroidering poetry onto fabric, turning her apartment into a three-dimensional collage, and creating daring costumes for Diaghilev’s *Ballets Russes.*

The diverse inspirations behind Delaunay’s work were also explored, from the highly personal approach to colour which harked back to her childhood in Russia, to the impact of her years in Spain and Portugal where she painted *The Orange Seller* [1915] and *Flamenco Singers* [1915–16].

The show also revealed the inspiration provided by modern technology throughout Delaunay’s career, from the Trans-Siberian Railway to the airplane, and from the Eiffel Tower to the electric light bulb. It also included her vast seven-metre murals *Motor*, *Dashboard* and *Propeller*, created for the 1937 International Exposition in Paris and never before shown in the UK.

Following her husband’s death in 1941, Sonia Delaunay’s work took on a more formal freedom, including rhythmic compositions in angular forms and harlequin colours, which in turn inspired geometric tapestries, carpets and mosaics. Delaunay continued to experiment with abstraction in the post-war era, just as she had done since its birth in the 1910s, becoming a champion for a new generation of artists and an inspiring figure for creative practitioners to this day. To illustrate the point, David Seidner wrote when interviewing her in Los Angeles in 1981:

A world of color would be ideal, where one could create emotions accordingly. We could live by impressions the way a blind man lives by touch. We could vivify or seduce, transmute or emote, the possibilities are endless. A world of colour so fine and pure, from the deepest innermost part of the human body to the pale washed evasiveness of the white of the human eye. We could live in a constant state of aura where every feeling manifested itself by color thus removing the lie from mankind ^1^

Sonia Delaunay took an early, perhaps the earliest, jump into non-objectivity where colour elicited from. Her work serves swift proof of a tenacious intensity with which she threw herself into her art, her life. She lived a philosophy of emotion; delving, gouging, tasting and creating. Through a direct communication with the gut, she relied on intuition rather than intelligence, as did men of stature such as Goethe. She strived to emulate such greatness.

It could be said Delaunay entered so far inside as to reach the womb. She returned not only to primitive sensibility in terms of the universal, but also in terms of woman, of motherhood. As early as 1911, Delaunay delved into the non-objective world. Remembering the peasant crafts of her native Russia, she juxtaposed pieces of fabric and fur to create a lyrical, one-dimensional assemblage of semi-geometric shapes; a primary research into the world of colour which was to dominate and dictate the theme of her life. Colour became her *leitmotif.*

She erected a scaffolding of new impressions to reach not upward but inward. This new language of feeling corresponded to the Futurist movement in Italy, the Constructivist in Russia and the *Blaue Reiter* in Germany. Sonia Delaunay is at the root, perhaps, of modern art.

Many people have too quickly made a distinction between fine and applied art. The fact that the first baby blanket profoundly influenced Robert Delaunay (after it he began his famous collages) is virtually neglected. She was no theoretician, thus she sought refuge in a more earthy medium. She applied her and her husband’s ideas of the ‘Simultaneous’ and ‘Pure Painting’ to a lamp shade of which she gave the name ‘Halo Depth;’ curtains: ‘Depth Movement;’ cushions: ‘Sec Movement Colors Depth’, ‘Astral’; goblets: ‘Moon Absinthe’, ‘Water Wine’, ‘Wine’.

She had already been involved herself in what one may call her *Fauve* period. Although she disliked Matisse, she was influenced by his transformation of the banal to the vivified via colour. From Gaugin, she took the organisation of flat coloured surfaces. And from Van Gogh, she took the intensity of colour. Although her paintings from this period are colourful, they are far from being ‘light’. In fact, one can trace the somber quality of pensive, intent studies, through her concept of depth being the inspiration for ‘pure painting’.

Married to Robert Delaunay, together they worked, collaborated and exchanged. The richness is legendary. Every night they walked along the Boulevard St. Michel where the gas lamps had just been replaced by electricity. They would return home and capture their impressions of colour, much the way Monet did, but in a new and different mode: a non-objective one. Together they walked to the Eiffel Tower, an edifice Robert believed to be the paragon of technology. In honour of their love, he did a small painting of the tower with an inscription on one side reading: ‘*Exposition Universelle … 1889 La Tour a l’Universe J’adresse*,’ and on the other: ‘*Mouvement Profondeur 1909 France*-*Russie*’.

1911–1912 marked the beginning of abstract art, and the beginning of the Delaunay’s experiments with colour. Sonia had just begun when she encountered the poetry of Blaise Cendrars. Its movement and association conjured further images of her rhythmic forms and her art was propagated. She made a book binding for his work and stated*: ‘Painting is a form of poetry, colors are words, their relations rhythms, the completed painting a completed poem’* She collaborated much with Cendrars and later made bindings for the works of Rimbaud, Walden, Apollinaire, Tzara and Mallarme.

On the Rue des Grand Augustins, the Delaunays held court. To their Thursday evening salons, came Cendrars, Apollinaire, Jean and Sophie Taeuber Arp and other painters, poets and musicians of the day. Groups would go together afterwards to the Bal Ballier in Montparnasse, a popular dance hall, where the Tango and the Fox Trot were all the rage. As early as 1912, Sonia Delaunay decorated her clothes with geometry and colour, freeing herself from flowers and frills. One may even call her the predecessor of art deco. Apollinaire wrote the following about her and her husband’s ensembles:

They do not burden themselves with the imitation of antiquated fashion, and since they want to be of their own time, they don’t innovate in the cut of cloth (in that they follow contemporary fashion), but rather they seek to influence it by employing new fabrics, infinitely varied with color. There is, for example, an outfit of M. Robert Delaunay: purple jakcet, beige vest, black trousers. Here is another: red coat, with a blue collar, red socks, yellow and black shoes, black trousers, green jacket, sky-blue vest, tiny red tie …

[And about Sonia Delauney]:

… purple dress, wide purple and green sash, and, under the jacket, a corsage divided into brightly colored zones, delicate or faded, where there is mixed an old rose, yellow-orange color, Nattier blue, scarlet, et cetera … appearing on different materials, so that wool cloth, taffeta, tulle, flannelette, watered silk, and peau de sale are juxtaposed … So much variety cannot escape notice. It transforms fantasy into elegance … ^2^

That fantastic elegance made its way through the First World War to the ‘*Exposition des Art Decoratifs’* in Paris, 1925. Sonia Delaunay’s collaboration with Jacques Heim celebrated her worldwide. Furs, automobiles, furniture, clothes, bags … nothing was out of her reach. She also did costumes, and those for Diaghilev remain among the Ballet Russe’s best. Cleopatra was swathed in circles stemming from the breast, giving costume the illusion of dance, the airy, ethereal step into another dimension. One critic said the dancers *‘set in motion costumes that already simulated motion …’*

The work by husband and wife continued earnestly as an exchange, an inspiration and a collaboration. The marriage of the Delaunays remained the most magical artistic merger. Sadly in 1941, Robert met an untimely death. With the Second World War, and Robert’s death, Sonia began to incorporate the colour black into her work. She relaised then that there are as many shades and textures of black as there are colours. Her work was interrupted when she devoted 10 years of her life to preserving her husband’s legend.

She worked until 90 but was arrested by failing health. She fell and was immobile for a long time. Her apartment in St. Germain des Pres was white and light filled. Ceilings soared, harbouring enormous tropical plants. There were works by her and her husband, and gifts from Jean Arp, Henri Laurens, Gilioli, Hans Hartung and others. The spirit of Sonia Delaunay was everywhere: on the floor in rugs, in a myriad of books and posters, catalogues, sketches, paintings. Sonia Delaunay was white on white: white hair with a cream complexion, few wrinkles and a prevailing sense of softness. In a sensual, raspy voice, she gave simple and direct answers in her last interview.

She died at 93.

## Footnotes

^1^ See www.bombmagazine.org/article/60 [Accessed 9 February 2016].

^2^ From Tate exhibition literature.

